# Survival and diagnostic age of 175 Taiwanese patients with mucopolysaccharidoses (1985–2019)

**DOI:** 10.1186/s13023-020-01598-z

**Published:** 2020-11-07

**Authors:** Hsiang-Yu Lin, Chung-Lin Lee, Chia-Ying Chang, Pao Chin Chiu, Yin-Hsiu Chien, Dau-Ming Niu, Fuu-Jen Tsai, Wuh-Liang Hwu, Shio Jean Lin, Ju-Li Lin, Mei-Chyn Chao, Tung-Ming Chang, Wen-Hui Tsai, Tzu-Jou Wang, Chih-Kuang Chuang, Shuan-Pei Lin

**Affiliations:** 1 Department of Medicine, MacKay Medical College, New Taipei City, Taiwan; 2 Department of Pediatrics, MacKay Memorial Hospital, No.92, Sec. 2 Chung-Shan North Road, Taipei, 10449 Taiwan; 3 Department of Medical Research, MacKay Memorial Hospital, 92 Chung-Shan N. Rd., Sec. 2, Taipei, 10449 Taiwan; 4 Nursing and Management, MacKay Junior College of Medicine, Taipei, Taiwan; 5 Department of Medical Research, China Medical University Hospital, China Medical University, Taichung, Taiwan; 6 Institute of Biomedical Sciences, MacKay Medical College, New Taipei City, Taiwan; 7 Department of Pediatrics, MacKay Memorial Hospital, Hsinchu, Taiwan; 8 Institute of Clinical Medicine, National Yang-Ming University, Taipei, Taiwan; 9 Department of Pediatrics, Kaohsiung Veterans General Hospital, Kaohsiung, Taiwan; 10 Department of Pediatrics, National Taiwan University Hospital, Taipei, Taiwan; 11 Department of Pediatrics, Taipei Veterans General Hospital, Taipei, Taiwan; 12 Department of Pediatrics, China Medical University Hospital, Taichung, Taiwan; 13 Department of Pediatrics, Chi Mei Medical Center, Tainan, Taiwan; 14 Department of Pediatrics, Chang Gung Memorial Hospital at Linkou, Taoyuan, Taiwan; 15 Department of Pediatrics, Changhua Christian Children’s Hospital, Changhua, Taiwan; 16 Department of Pediatric Neurology, Changhua Christian Children’s Hospital, Changhua, Taiwan; 17 School of Medicine, Kaohsiung Medical University, Kaohsiung, Taiwan; 18 Department of Pediatrics, Chang Gung Memorial Hospital, Kaohsiung, Taiwan; 19 College of Medicine, Fu-Jen Catholic University, Taipei, Taiwan; 20 Department of Infant and Child Care, National Taipei University of Nursing and Health Sciences, Taipei, Taiwan

**Keywords:** Early diagnosis, Mucopolysaccharidosis, Newborn screening, Survival, Taiwan

## Abstract

**Background:**

Mucopolysaccharidoses (MPSs) are a group of inherited metabolic diseases, which are characterized by the accumulation of glycosaminoglycans, and eventually lead to the progressive damage of various tissues and organs.

**Methods:**

An epidemiological study of MPS in Taiwan was performed using multiple sources. The survival and diagnostic age for different types of MPS between 1985 and 2019 were evaluated.

**Results:**

Between 1985 and 2019, there were 175 patients diagnosed with MPS disorders in the Taiwanese population, with a median diagnostic age of 3.9 years. There were 21 (12%), 78 (45%), 33 (19%), 32 (18%) and 11 (6%) patients diagnosed with MPS I, II, III, IV and VI, respectively, with median diagnostic ages of 1.5, 3.8, 4.7, 4.5 and 3.7 years, respectively. Diagnosis of MPS patients was significantly earlier in recent decades (*p* < 0.01). Pilot newborn screening programs for MPS I, II, VI, IVA, and IIIB were progressively introduced in Taiwan from 2016, and 48% (16/33) of MPS patients diagnosed between 2016 and 2019 were diagnosed by one of these screening programs, with a median diagnostic age at 0.2 years. For patients born between 2016 and 2019, up to 94% (16/17) were diagnosed with MPS via the newborn screening programs. At the time of this study, 81 patients had passed away with a median age at death of 15.6 years. Age at diagnosis was positively correlated with life expectancy (*p* < 0.01). Life expectancy also significantly increased between 1985 and 2019, however this increase was gradual (*p* < 0.01).

**Conclusions:**

The life expectancy of Taiwanese patients with MPS has improved in recent decades and patients are being diagnosed earlier. Because of the progressive nature of the disease, early diagnosis by newborn screening programs and timely implementation of early therapeutic interventions may lead to better clinical outcomes.

## Introduction

Mucopolysaccharidoses (MPSs) are a group of inherited metabolic diseases, which are a result of deficiencies in specific lysosomal enzymes that are required for the degradation of glycosaminoglycans (GAGs). It is known that there are eleven enzymes involved in the catabolism of chondroitin sulfate (CS), dermatan sulfate (DS), heparan sulfate (HS), keratan sulfate (KS) and hyaluronic acid. Deficiency in a specific enzyme results in the accumulation of undegraded GAGs, which causes progressive damage to cells, tissues and organs [1]. The clinical manifestations of MPS include developmental delay, coarse face, corneal clouding, hearing impairment, adenotonsillar hypertrophy, airway obstruction, obstructive sleep apnea, pulmonary function impairment, cardiac disease, hepatosplenomegaly, umbilical and inguinal hernias, growth retardation, kyphoscoliosis, skeletal deformities (dysostosis multiplex), and joint contractures. Chronic and progressive signs and symptoms may emerge in patients from early to late childhood or even in early adulthood. MPS has a broad spectrum of clinical presentations with varied severity and prognosis for different types and individuals [2–6]. MPS disorders are autosomal recessive, except for MPS II (Hunter syndrome), which has X‐linked inheritance and mainly affects males. The prevalence of MPS in different populations ranges from 1.35 (South Korea) to 4.5 (The Netherlands) per 100,000 live births [7–9]. In a Taiwanese study, it was estimated to be 2.04 per 100,000 live births between 1984 and 2004 [10].

The clinical presentations of the different types of MPS disorders can be categorized into three groups in accordance with the type of GAG accumulation. The “visceral” group is caused by DS accumulation and includes patients with MPS I, II, VI, and VII manifesting coarse facial features, corneal clouding, hearing loss, adenotonsillar hypertrophy, upper airway obstruction, heart disease, hepatosplenomegaly, joint stiffness, skeletal deformities, and short stature. The “neurodegenerative” group is caused by HS accumulation and includes patients with MPS IIIA, B, C, and D, MPS I (Hurler syndrome), and the severe form of MPS II manifesting hyperactivity, cognitive decline, and behavioral disturbances. The “skeletal” group is caused by KS accumulation and includes patients with MPS IV manifesting odontoid hypoplasia, joint laxity, genu valgum, skeletal dysplasia, and extreme short stature [1, 11]

Hematopoietic stem cell transplantation (HSCT) and enzyme replacement therapy (ERT) are the main treatments for MPS disorders. HSCT is now the only therapy to prevent progressive neurodegenerative disorders in MPS I. There are only limited data available with partial benefits for the other types of MPS [12]. ERT is currently available for MPS I, II, IVA, VI, and VII, and it has been demonstrated to substantially reduce urinary GAG levels and significantly improve endurance, physiological activities, joint mobility, and quality of life [13–20]. Additional therapies for MPS disorders are currently in clinical trials, and these include chaperone therapy, substrate reduction therapy, and gene therapy [21]. Previous reports have shown that early treatment may lead to better clinical outcomes [22, 23].

Early diagnosis for MPS through newborn screening programs when it is asymptomatic, could allow for more timely and appropriate treatment plans for these patients, as well as more comprehensive genetic counseling for the family members. Since pilot newborn screening programs were introduced in Taiwan for MPS I, II, VI, IVA, and IIIB sequentially since 2016, the median age of a confirmed diagnosis for MPS could be as low as 0.2 years. At this age patients are asymptomatic and it is much earlier than the current median diagnostic age of 4.3 years in Taiwan, by which time patients are symptomatic [5, 24–27]. In addition, information regarding survival rates and diagnostic age for different types of MPS in a single population is lacking in the literature. The purpose of the current study was to retrospectively collect and analyze data on life expectancy and diagnostic age as recorded on the medical charts of Taiwanese MPS patients who were diagnosed between 1985 and 2019. This could help us better understand the natural progression of this disease, as well as the effect of newborn screening programs for MPS.

## Materials and methods

### Study population

To obtain as much data as possible on all patients diagnosed with MPS in Taiwan between 1985 and 2019, the following sources were utilized: (1) Membership list of the Taiwan MPS Society (patient support group); (2) Medical records from 10 medical centers in Taiwan, including MacKay Memorial Hospital, Taipei; Kaohsiung Veterans General Hospital; National Cheng Kung University Hospital, Tainan; China Medical University Hospital, Taichung; National Taiwan University Hospital, Taipei; Taipei Veterans General Hospital; Chi Mei Medical Center, Tainan; Changhua Christian Children’s Hospital, Changhua; Chang Gung Memorial Hospital, Kaohsiung; and Chang Gung Memorial Hospital, Taoyuan; (3) Laboratory records from the Department of Medical Research, MacKay Memorial Hospital, Taipei, Taiwan; (4) Records from the Taiwan Foundation for Rare Disorders; and (5) Records from the Health Promotion Administration, Ministry of Health and Welfare, Taiwan.

The diagnosis of the type of MPS was confirmed by a specific enzyme activity assay in serum, leukocytes and/or skin fibroblasts, two-dimensional electrophoresis of urinary GAGs, quantitative determination of DS, HS, KS, and CS using liquid chromatography-mass spectrometry (LC–MS/MS), and/or identification of the pathogenic mutations [28–30]. MPS I patients were classified into three syndromes according to their clinical manifestations: Hurler syndrome (severe), Hurler–Scheie syndrome (intermediate), and Scheie syndrome (attenuated). For patients with MPS II, the severe form was defined according to the existence of cognitive impairment compared with the mild form (without cognitive impairment). MPS type, gender, date of birth, diagnostic age, and date of death (if applicable) for these patients were retrospectively reviewed. Institutional Review Board/Ethics Committee approval was obtained for all participating centers. Written informed consent was obtained from each patient, or their parents or legal representative. For patients who died before this study entry, consent was obtained from patients’ families. All patients’ information was managed according to national data protection standards.

### Data and statistical analysis

The results of descriptive statistics are shown as the mean ± standard deviation or median value unless otherwise indicated. The association between age at diagnosis and year of birth, and the year of death and age at death for the MPS patients were assessed using Pearson’s correlation coefficient (*r*). Testing for statistical significance was performed using Fisher’s *r*–*z* transformation. All statistical analyses were performed using SPSS version 11.5 (SPSS Inc., Chicago, Illinois, USA), and differences with *p* < 0.05 were considered to indicate a statistically significant difference.

## Results

**Fig. 1:**
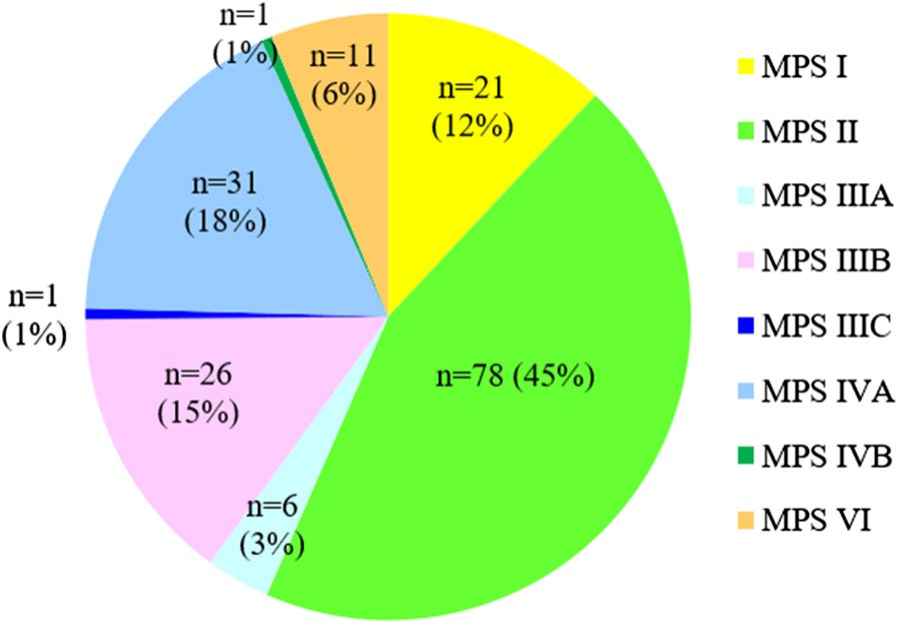
Number and percentage of different types of MPS in Taiwan from 1985 to 2019 (n = 175)

**Fig. 2:**
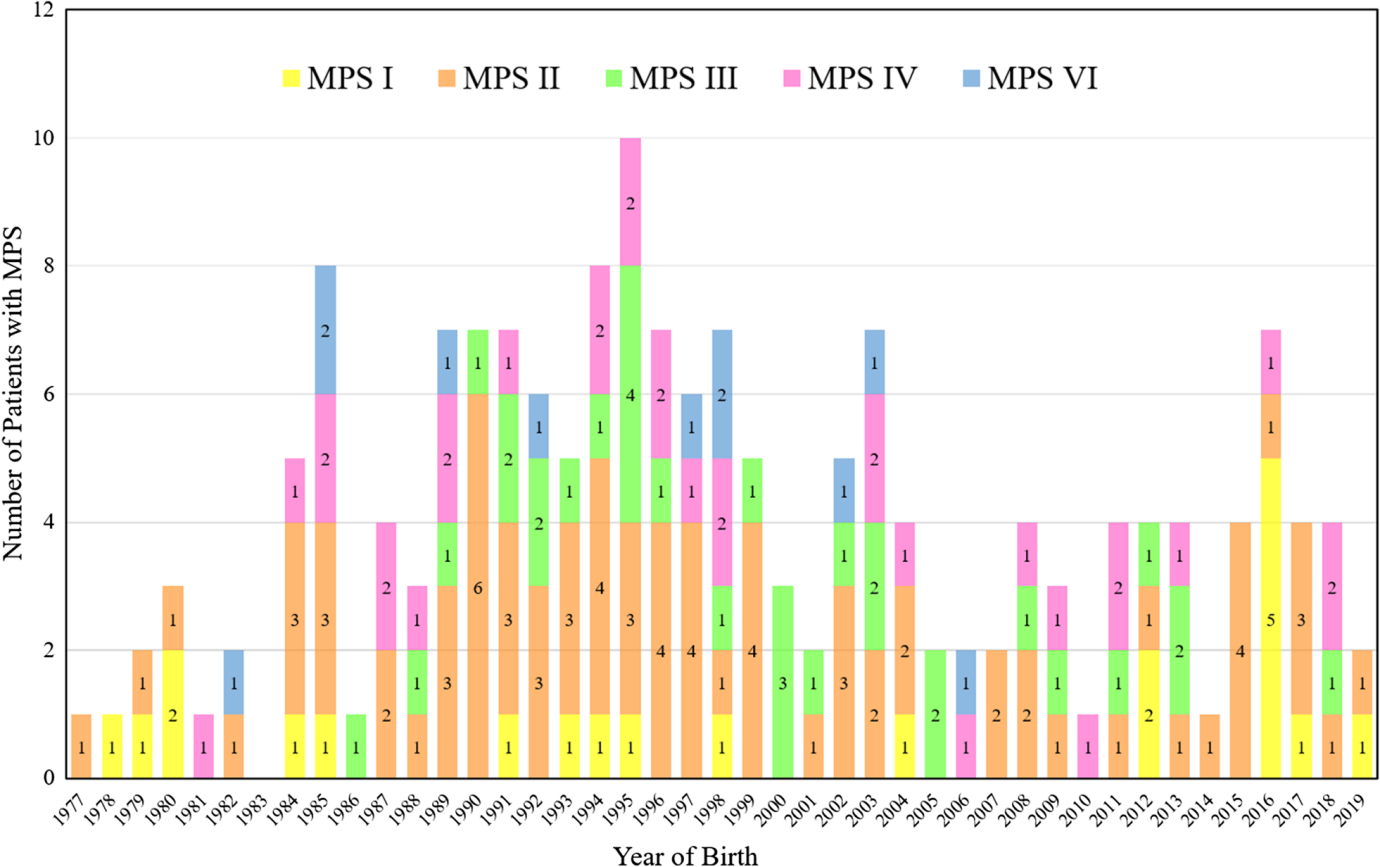
MPS type and year of birth for 175 Taiwanese patients

**Fig. 3:**
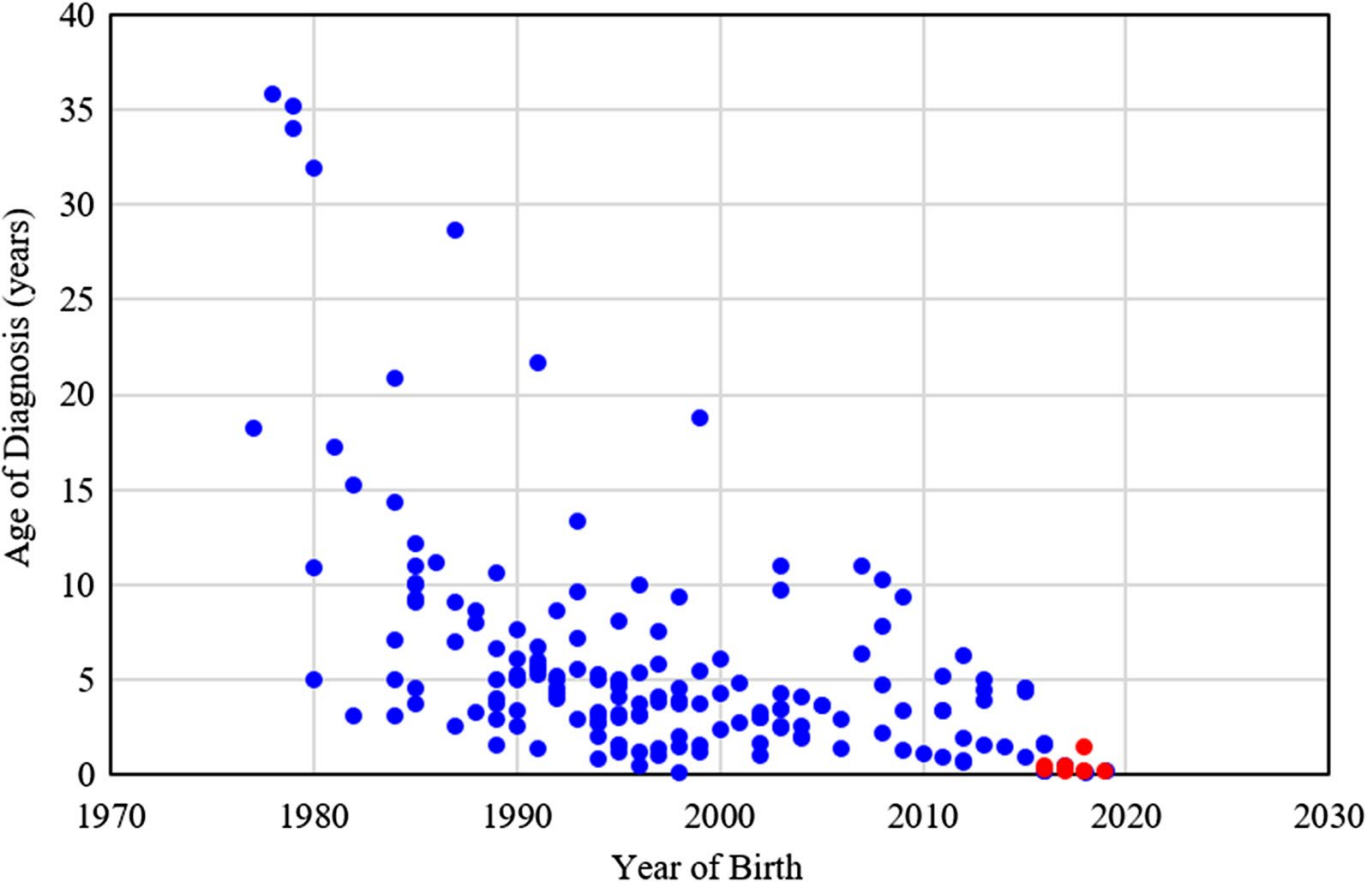
Negative correlation between year of birth and age of diagnosis for 175 patients with MPS (*r* = − 0.537, *p* < 0.01). Blue dots represent diagnosis from clinical indications, and red dots represent diagnosis from the newborn screening programs that were started in Taiwan from 2016

**Fig. 4:**
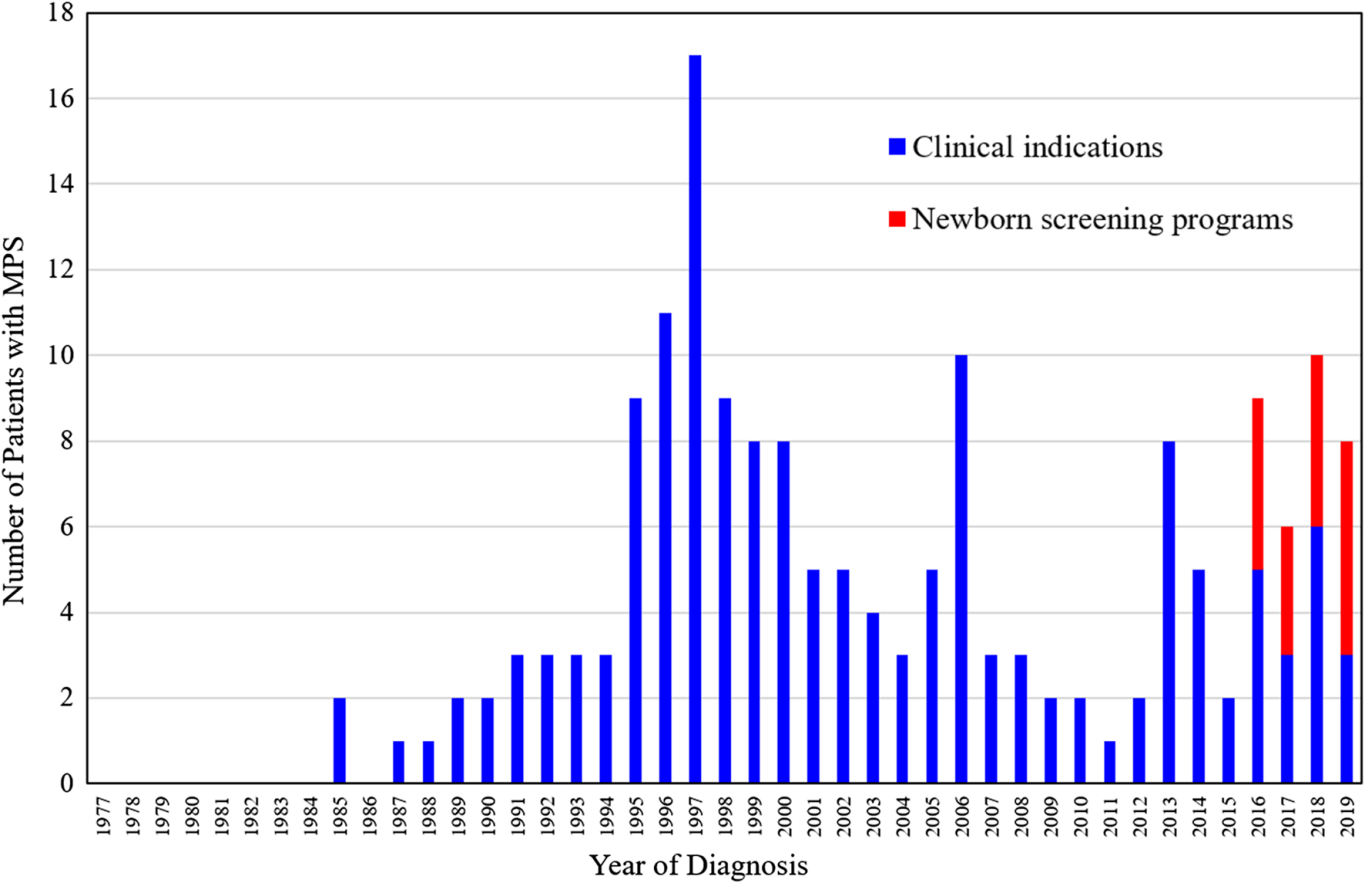
The number of patients diagnosed with MPS each year and the origin of their diagnosis. Blue = clinical indications; Red = the newborn screening programs started in Taiwan from 2016

**Fig. 5:**
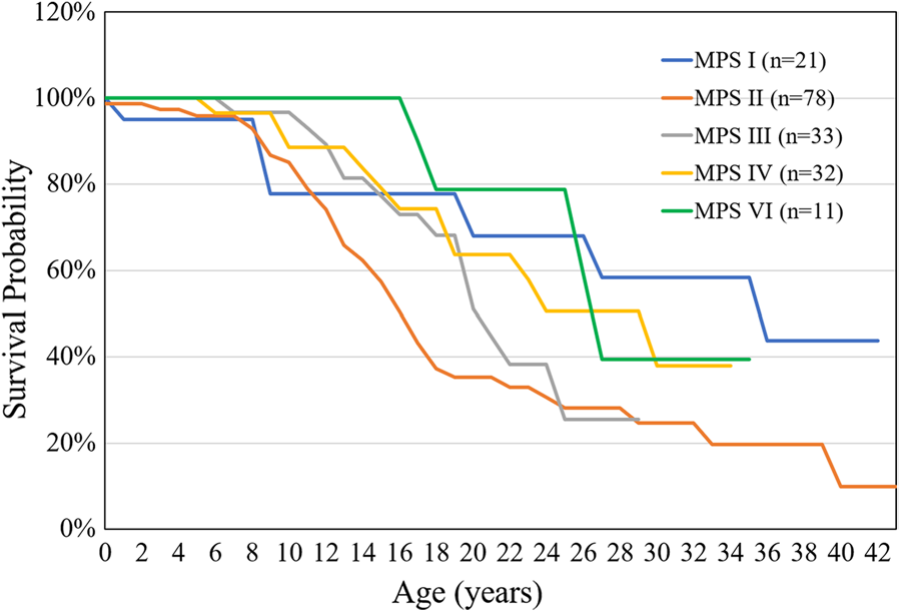
Kaplan–Meier survival curve for 175 patients with different types of MPS

**Fig. 6:**
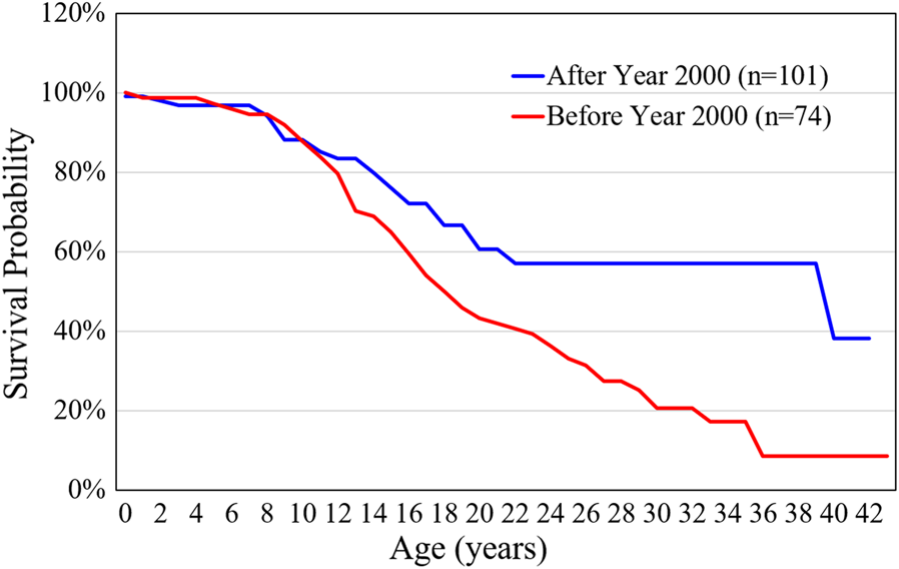
Kaplan–Meier survival curves for 175 patients diagnosed with MPS before and after the year 2000

**Fig. 7:**
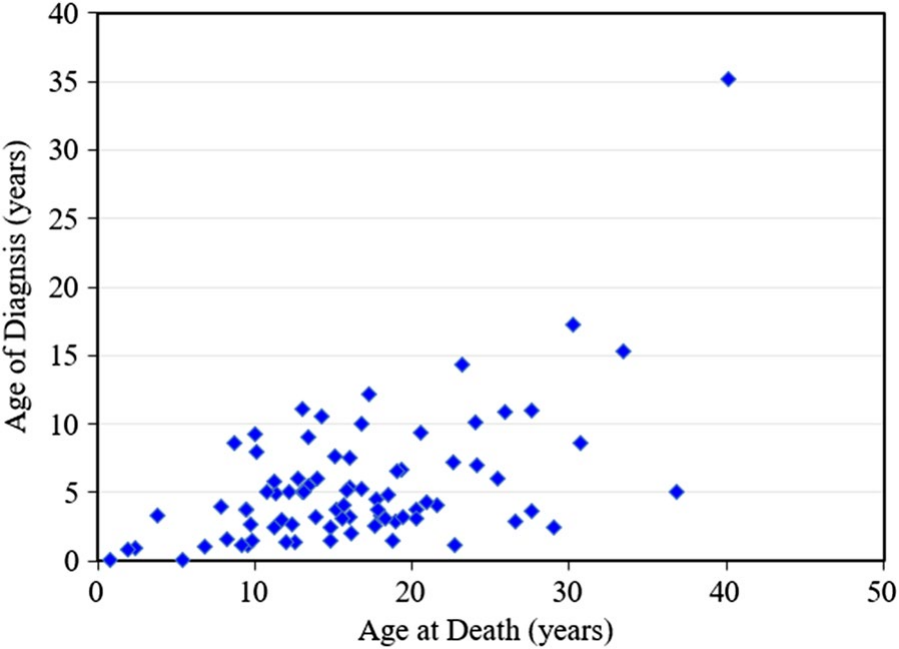
Positive correlation between age at death and age of diagnosis for 81 patients with MPS (*r* = 0.560, *p* < 0.01)

**Fig. 8:**
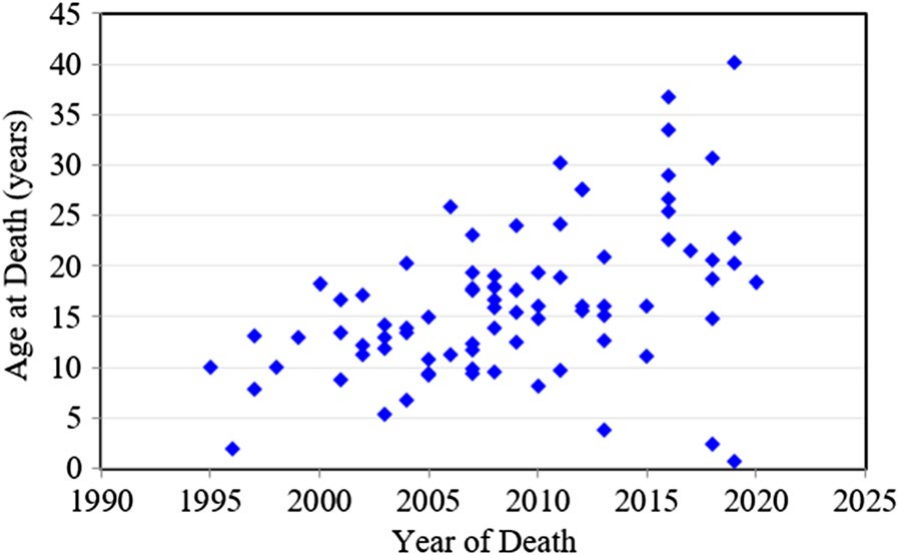
Positive correlation between the year of death and age at death for 81 patients with MPS (*r* = 0.459, *p* < 0.01)

Between 1985 and 2019, there were 175 patients with MPS disorders diagnosed in the Taiwanese population. There were 21 (12%), 78 (45%), 33 (19%), 32 (18%) and 11 (6%) patients diagnosed with MPS I, II, III, IV and VI, respectively. Among the 33 patients with MPS III, MPS IIIB was the largest group (n = 26), followed by MPS IIIA (n = 6) and MPS IIIC (n = 1). Among the 32 patients with MPS IV, the majority had MPS IVA (n = 31) and one patient had MPS IVB (Fig. 1). Figure 2 shows the MPS type of the 175 patients and their year of birth. The oldest patient in this cohort had MPS II and was born in 1977. Between 1977 and 2019, there were individuals born with MPS every year except in 1983. Table 1 reports the mean and median age at diagnosis and at death for patients with different types of MPS. The median diagnostic age for all 175 patients was 3.9 years. Among the different types of MPS, the earliest median diagnostic age was 1.5 years for MPS I, followed by MPS VI (3.7 years), MPS II (3.8 years), MPS IV (4.5 years), and MPS III (4.7 years). Patients were diagnosed with MPS significantly earlier in recent decades (*p* < 0.01) (Fig. 3). Figure 4 shows the number of patients who were diagnosed with MPS each year and whether this diagnosis was due to clinical indications or the newborn screening programs. Before the implementation of Taiwanese National Health Insurance in 1995, the number of diagnoses for MPS was no more than three patients a year, however, after 1995, the number increased to 8–17 patients every year between 1995 and 2000. As the pilot newborn screening programs for MPS I, II, VI, IVA, and IIIB were progressively implemented in Taiwan from 2016, 48% (16/33) of MPS patients diagnosed between 2016 and 2019 were referred from newborn screening programs and had a median diagnostic age of 0.2 years. This was notably lower than the median diagnostic age of 4.3 years for the other 159 patients who were diagnosed due to clinical indications. For individuals born between 2016 and 2019 who were diagnosed with MPS, 94% (16/17) were referred from the newborn screening programs. Only one patient born in 2016 was diagnosed with MPS IVA at 1.6 years due to clinical indications. Among these 175 patients with MPS, seventy-seven patients had ever received ERT, and 8 patients had ever received HSCT. The median ages at the start of receiving ERT and HSCT were 9.8 and 4.0 years, respectively. Of the 16 MPS patients diagnosed from newborn screening programs, intravenous ERT was started in 9 (patient numbers 1–4, 6–8, 15, 16) following the diagnosis. Patient numbers 2, 3 and 7 also received HSCT at 0.9, 0.6 and 1.5 years of age, respectively. Patient 2 received HSCT at 0.9 years old. He had ever developed some complications after HSCT with the manifestations of severe edema, partial graft-versus-host disease, and liver function impairment, but the condition was managed properly thereafter. He had normal blood leukocyte iduronate-2-sulfatase activity of 13.78 μmol/gm protein/4 h (reference range 12.89–131.83) at 1.2 years old. Patient 3 received HSCT at 0.6 years old, however, he died at 0.8 years old due to infection and sepsis. Patient 7 had good outcome after receiving HSCT at 1.5 years old. He had 95% engraftment and normal blood leukocyte iduronate-2-sulfatase activity of 69.51 μmol/gm protein/4 h (reference range 12.89–131.83) at 2.7 years old. The other seven patients had regular follow-ups at the clinic every 6-months for observation of emerging MPS symptoms. At the time of this study, these patients have not developed significant symptoms (Table 2). In the current study, 81 patients passed away at a median age of 15.6 years. There were 6, 46, 15, 11 and 3 patients with MPS I, II, III, IV and VI who passed away, with a median survival age of 15, 14.1, 18.5, 16.8 and 26.6 years, respectively (Table 1). Among the 6 patients with MPS I, two had Hurler syndrome and a median survival age of 5.9 years, and 4 had Hurler–Scheie syndrome with a median survival age of 23.9 years. Among the 46 patients with MPS II, 36 had the severe form with a median survival age of 13.1 years, and 10 had the mild or intermediate form with a median survival age of 18.2 years (Table 3). Among these 81 patients who passed away, fourteen patients had ever received ERT with the mean survival age of 19.9 years, compared with 67 ERT-naive patients with the mean survival age of 15.7 years. ERT seems to have beneficial effects on the survival of the MPS patients. Figure 5 shows the Kaplan–Meier survival curves for the different types of MPS. Figure 6 shows the Kaplan–Meier survival curves for patients diagnosed with MPS before and after the year 2000. The survival probability was similar between these two groups before 10 years of age. However, the survival probability was higher for patients diagnosed after the year 2000 compared with those diagnosed before the year 2000 after their teenage years. The gap between these two groups gradually increased as the patients got older. In addition, life expectancy was significantly positively correlated with the age at diagnosis (*p* < 0.01) (Fig. 7). The patient’s longevity also significantly increased between 1985 and 2019, however this increase was gradual (*p* < 0.01) (Fig. 8).

**Table 1:** The mean and median age of diagnosis and at death for patients with different types of MPS

MPS type	I	II	III	IV	VI	All
Age of diagnosis (years)						
n	21	78	33	32	11	175
Mean (SD)	7.7 (12.1)	5.4 (5.6)	4.9 (2.6)	5.8 (5.7)	3.9 (2.1)	5.6 (6.2)
Median	1.5	3.8	4.7	4.5	3.7	3.9
Age at death (years)						
n	6	46	15	11	3	81
Mean (SD)	17.6 (13.0)	15.0 (7.3)	18.1 (5.9)	17.3 (6.9)	24.2 (5.1)	16.4 (7.6)
Median	15.0	14.1	18.5	16.8	26.6	15.6

*MPS* mucopolysaccharidosis, *SD* standard deviation

**Table 2:** The demographic data of 17 Taiwanese patients with MPS born between 2016 and 2019

No	Year of birth	Year of diagnosis	Age of diagnosis (years)	MPS type	Referring origin	Management
1	2019	2019	0.2	I	NBS	ERT
2	2019	2019	0.2	II	NBS	ERT + HSCT
3	2018	2019	0.1	II	NBS	ERT + HSCT
4	2018	2018	0.1	IVA	NBS	ERT
5	2018	2019	0.1	IIIB	NBS	Regular follow-up
6	2018	2019	1.4	IVA	NBS	ERT
7	2017	2017	0.1	II	NBS	ERT + HSCT
8	2017	2018	0.2	II	NBS	ERT
9	2017	2017	0.4	I	NBS	Regular follow-up
10	2017	2018	0.5	II	NBS	Regular follow-up
11	2016	2016	0.1	I	NBS	Regular follow-up
12	2016	2016	0.1	I	NBS	Regular follow-up
13	2016	2016	0.1	II	NBS	Regular follow-up
14	2016	2016	0.2	I	NBS	Regular follow-up
15	2016	2017	0.4	I	NBS	ERT
16	2016	2018	1.6	I	NBS	ERT
17	2016	2017	1.6	IVA	Clinical indication	ERT

*MPS* mucopolysaccharidosis, *NBS* newborn screening, *ERT* enzyme replacement therapy, *HSCT* hematopoietic stem cell transplantation

**Table 3:** The mean and median age at death for patients with different subtypes of MPS I and MPS II

Age at death (years)	MPS I			MPS II		
	I (H)	I (H/S)	All	II (S)	II (M) and II (I)	All
n	2	4	6	36	10	46
Mean (SD)	5.9 (5.6)	23.4 (11.7)	17.6 (13.0)	13.0 (5.5)	22.0 (9.0)	15.0 (7.3)
Median	5.9	23.9	15.0	13.1	18.2	14.1

*MPS* mucopolysaccharidosis, *MPS I (H)* Hurler syndrome, *MPS I (H/S)* Hurler–Scheie syndrome, *MPS II (S)* severe form, *MPS II (M)* mild form, *MPS II (I)* intermediate form, *SD* standard deviation

## Discussion

To the best of our knowledge, this is the first multicenter study to analyze the survival and diagnostic age of patients with various types of MPS in a countrywide population across a 35 year period. We found that the life expectancy and diagnostic age of patients with MPS had improved in recent decades. In Taiwan, the National Health Insurance program was implemented in 1995, the Rare Diseases and Orphan Drugs Act was passed in 2000, and countrywide MPS newborn screening programs were started in 2016 with a median diagnostic age of 0.2 years. Thus, it is possible that the observed improvement in life expectancy may be due to the timely referral of MPS patients to specialists and the early administration of multidisciplinary care, as well as medical advancements in ERT and HSCT.

For Taiwanese patients with MPS in the current cohort, MPS II was the most prevalent subgroup (45%), followed by MPS III (19%), MPS IV (18%), MPS I (12%), and MPS VI (6%). To date, there has been no confirmed patient with MPS VII in Taiwan. The predominance of MPS II in Taiwan (45%) is consistent with other Asian countries, including China (47.4%), Japan (55%), and South Korea (54.6%). However, in most Caucasian populations, MPS III or MPS I is the most prevalent MPS type [9]. The founder effect may explain the differences in MPS type prevalence across various ethnic populations.

The median diagnostic age for patients with all types of MPS was 3.9 years. In the current study, children under 5 years of age, especially those who were diagnosed through the newborn screening programs and did not have significant clinical manifestations, were not categorized into MPS I and II subgroups (severe vs. mild), due to the difficulty in determining mental development status. For MPS I, the data from the MPS I Registry (n = 891) reported by D’Aco et al*.* [31] indicated that the median diagnostic age was 0.8 years for Hurler syndrome, 3.8 years for Hurler–Scheie syndrome, and 9.4 years for Scheie syndrome. In the present study, the median diagnostic age was 1.5 years for all MPS I patients (n = 21). Parini et al. [32] reported that the median diagnostic age for MPS II was 3.0 years (n = 316) for those with the severe form, and 3.8 years for those with the attenuated form (n = 320). Similarly, the median diagnostic age for all MPS II patients (n = 78) was 3.8 years in the current study. Truxal et al. [33] reported that the mean diagnostic age for MPS III was 3.4 years (n = 25). In the current study the mean diagnostic age for MPS III was 4.9 years (n = 33). Data from the International Morquio A Registry (n = 311) reported by Montaño et al. [34] revealed a mean diagnostic age of 4.7 years for MPS IV, which was supported by our results (5.8 years; n = 32). Information on the median diagnostic age for MPS VI patients was lacking in the literature. In the current cohort, the median diagnostic age was 3.7 years (n = 11).

Since MPS is a rare, progressive, multisystemic disease with insidious initial signs and symptoms, making an early diagnosis can be challenging for first-line general practitioners [35]. Newborn screening programs could help provide an early diagnosis for MPS at an asymptomatic age, thus allowing for more timely and appropriate treatment for these patients. Since the pilot newborn screening programs for MPS I, II, VI, IVA, and IIIB were progressively implemented in Taiwan from 2016, up to 94% (16/17) of MPS patients born between 2016 and 2019 have been diagnosed via the programs, with a median diagnostic age of 0.2 years. This is notably lower than the median age at diagnosis of 4.3 years for the other 159 patients diagnosed via clinical indications during the same time period. In the present study, 9 of the 16 patients diagnosed via the newborn screen programs received ERT following their diagnosis; payments for this were reimbursed by the Taiwanese National Health Insurance program according to international standards. In addition, 3 MPS II patients also received HSCT. Following a comprehensive explanation of the progressive nature of MPS and the importance of the early initiation of ERT or HSCT before the occurrence of irreversible organ damage, the parents of the other seven patients elected for their children to receive regular follow-ups every 6 months at the clinic to check for any emerging symptoms of MPS. Although ERT and HSCT cannot cure the disease, they can improve or alleviate its natural progression, and better outcomes may be associated with the early administration of these treatments [21–23]. Since survival time and the natural disease course may be significantly altered in patients who receive early ERT and HSCT, longer follow-up periods are warranted to observe the efficacy of these treatments in children with MPS who are diagnosed early via the newborn screen programs.

The natural disease course and overall prognosis vary among different MPS types and there is a wide spectrum of clinical severity [2, 32, 36–44]. Data from the MPS I Registry (n = 180) reported by D’Aco et al. [31] revealed that the median age at death for all MPS I patients was 5.1 years (range 0.4–46.6). In the present study, the median age at death was 15.0 years (n = 6). For MPS II, the most severely affected patients with MPS II usually only survive until their second decade of life, however, less severely affected patients may survive until their fifth or sixth decade of life [36, 37]. The Hunter Outcome Survey (HOS) data (n = 129) reported by Jones et al*.* [38] showed that the median age of death was 13.4 years for MPS II. In our study, the median age of death was similar at 14.1 years (n = 46). We also found that the MPS II patients with cognitive impairment had a shorter life span than those without cognitive impairment (13.1 years vs. 18.2 years), which was consistent with previous reports [38, 39]. For MPS III, Lavery et al. [40] reported that the mean age at death between 1977 and 2007 was 15.2, 18.9 and 23.4 years for patients in the UK with MPS IIIA (n = 84), MPS IIIB (n = 24), and MPS IIIC (n = 5), respectively. Similarly, the present study found that the mean age at death for MPS III (n = 15) was 18.5 years for patients in Taiwan. For MPS IV, Lavery and Hendriksz [42] reported that the mean age at death was 25.3 years for 27 patients with MPS IVA in the UK between 1975 and 2010. The present study showed that the mean age at death was 17.3 years for patients with MPS IVA (n = 11). For MPS VI, the MPS VI Survey Study reported by Giugliani et al. [44] showed that the mean age at death for 17 ERT patients and 7 ERT-naive patients was 22.9 and 19.2 years, respectively. The current study showed that the mean age at death for MPS VI (n = 3) was 24.2 years, including two ERT patients who died at 26.6 and 27.6 years, respectively, and one ERT-naive patient who died at 18.3 years. Our results also revealed that ERT may lengthen the life expectancy of patients with MPS VI.

In the current study, the age at diagnosis was positively correlated with life expectancy (*p* < 0.01). A possible explanation for this might be that MPS has a broad spectrum of disease severity, and that patients with a milder form may manifest more subtle clinical signs and symptoms leading to a delay in their diagnosis or even a misdiagnosis with other disorders, as well as longer survival.

In the current study, the life expectancy for patients significantly increased between 1985 and 2019, however this increase was gradual (*p* < 0.01). In keeping with this finding, Jones et al. [38] reported in the HOS that the median age at death was significantly lower in patients who died in or before 1985 compared with those who died after 1985 (11.3 vs. 14.1 years, *p* < 0.001). Sohn et al. [39] also reported that patients who died after 2005 lived significantly longer than those who died before 2005 (19.4 vs. 11.4 years, *p* < 0.05). This may reflect advancements in medical care, early diagnosis, and appropriate treatment for MPS patients over the past few decades.

Taiwan is a geographically isolated island with a relatively stable population. The National Health Insurance program was carried out in Taiwan in 1995, which lessened family financial burdens for health care and provided the rights of the public to have equal-access to medical care. This background condition may explain the status that before the implementation of Taiwanese National Health Insurance in 1995, the number of diagnoses for MPS was no more than three patients a year, however, after 1995, the number increased to 8–17 patients every year between 1995 and 2000. The Rare Diseases and Orphan Drugs Act was also implemented in Taiwan in year 2000. Under these regulations, patients suspected of MPS disorders are referred to specialists for further evaluations, and the costs are fully subsidized by the National Health Insurance. Since there were constant numbers of diagnoses for MPS made every year throughout the study period, it is unlikely that a significant number of cases were lost because of lacking ascertainment. Meanwhile, our hospital is the major diagnostic center for MPS disorders in Taiwan [10]. Most of the patients clinically suspected to have an MPS disorder are referred to our hospital for further confirmation. Almost all MPS patients identified in Taiwan become members of the Taiwan MPS Society. They also registered in the records of the Taiwan Foundation for Rare Disorders and the Health Promotion Administration, Ministry of Health and Welfare, Taiwan. All MPS patients in this cohort had medical records in these 10 medical centers. As a result, we have confidence that few diagnoses were missed, and our survey reflects a reliable estimate of the Taiwanese patients with MPS. However, we are aware of that few patients could be missed are “less coarse”, but may be very ill from respiratory and cardiovascular complications in infancy; consequently, not identified by primary care providers and died young before the diagnosis was made.

Because the newborn screening programs for various types of MPS were still self-paid items in Taiwan, not all newborns received these screening programs. The screening rate was around 80–90% in the recent years. The newborn screening programs for various types of MPS were executed by Neonatal Screening Centers of The Chinese Foundation of Health, Taipei Institute of Pathology, and National Taiwan University Hospital. MacKay Memorial Hospital and National Taiwan University Hospital are the only referring medical centers for confirmative diagnosis in these programs [24–27]. As a result, we are confident that all flag-positive patients are followed up in this cohort. Although there may be a few false-negative MPS cases and missed by the newborn screening programs, however, the actual number should be very low.

### Limitations

As a retrospective, multicenter study, some medical records were missing and were not available at the time of this study. The relatively small sample size for each type of MPS in this cohort reflects the rare nature of this genetic disorder, and the degree of disease severity was quite wide. Consequently, studies with a larger study cohort and a longer follow-up period are warranted to validate these findings.

## Conclusion

Life expectancy for Taiwanese MPS patients has improved in recent decades, and patients are being diagnosed earlier. In Taiwan, the National Health Insurance Program was implemented in 1995, the Rare Diseases and Orphan Drugs Act was passed in 2000, and countrywide MPS newborn screening programs were initiated from 2016 (with a median diagnostic age of 0.2 years). So, it is possible that the early referral of patients to specialists and improvements in multidisciplinary care, as well as medical advancements in ERT and HSCT in recent decades are the underlying reasons for this trend. Because of the progressive nature of the disease, early diagnosis via newborn screening programs and timely initiation of ERT or HSCT before the occurrence of irreversible organ damage, may lead to better clinical outcomes. The findings of the current study could also serve as baseline data for the analysis of the long-term effects of ERT and HSCT on patients with MPS, and could help develop quality of care strategies.

## Data Availability

Not applicable. There are no other supporting data and materials since all of them are in this article.

## Abbreviations

MPS: Mucopolysaccharidosis
GAGs: Glycosaminoglycans
CS: Chondroitin sulfate
DS: Dermatan sulfate
HS: Heparan sulfate
KS: Keratan sulfate
HSCT: Hematopoietic stem cell transplantation
ERT: Enzyme replacement therapy
LC–MS/MS: Liquid chromatography/tandem mass spectrometry
